# *Mitrephoramonocarpa* (Annonaceae): a new species from Surat Thani Province, Peninsular Thailand

**DOI:** 10.3897/phytokeys.121.34271

**Published:** 2019-05-07

**Authors:** Richard M.K. Saunders, Piya Chalermglin

**Affiliations:** 1 School of Biological Sciences, The University of Hong Kong, Pokfulam Road, Hong Kong, China The University of Hong Kong Hong Kong China; 2 Agricultural Technology Department, Thailand Institute of Scientific & Technological Research, 35 Technopolis, Liap Khlong Ha Road, Khlong Luang District, Pathum Thani Province 12120, Thailand Thailand Institute of Scientific & Technological Research Bangkok Thailand

**Keywords:** Annonaceae, *
Mitrephora monocarpa
*, Thailand, new species

## Abstract

A new species, *Mitrephoramonocarpa* R.M.K.Saunders & Chalermglin, **sp. nov.** (Annonaceae), is described from Surat Thani Province in Peninsular Thailand. It resembles two other species, *M.alba* Ridl. and *M.keithii* Ridl., with which it is broadly sympatric, but can be distinguished by its solitary flowers (not borne in thyrsoid inflorescences as in most other *Mitrephora* species), single carpel per flower (and hence single monocarp per fruit) and the lack of a monocarp stipe. A key to the nine *Mitrephora* species in Thailand is provided.

## Introduction

*Mitrephora* (Blume) Hook.f. & Thomson (Annonaceae subfam. Malmeoideae tribe Miliuseae; Chatrou et al. 2012; Guo et al. 2017) is a genus of small to medium-sized trees widespread in lowland tropical forests in Southeast Asia. In the most recent taxonomic monograph of the genus, Weerasooriya and Saunders (2010) recognised 47 species (with an additional species subsequently recorded from Borneo: Okada 2014). The genus is likely to be monophyletic (Weerasooriya and Saunders 2010; Guo et al. 2017) and is characterised by extra-axillary (rarely terminal) cymose inflorescences composed of generally small, trimerous flowers with a whorl of sepals and two whorls of petals. The outer petals are larger than the inner and are free and spreading, whereas the inner petals are clawed-rhombic and apically connivent over the reproductive organs, forming a small mitriform dome (a ‘type III’ chamber, sensu Saunders 2010); basal apertures between the inner petal claws enable entry to the floral chamber by pollinators (unknown for most species but reported to be small nitidulid beetles in *M.heyneana* (Hook.f. & Thomson) Thwaites: Weerasooriya and Saunders 2010). The flowers are invariably hermaphroditic, with numerous stamens with an apical connective that is expanded to cover the top of the thecae and a variable number of unfused carpels. These carpels develop into free monocarps after fertilisation.

There are currently eight *Mitrephora* species recorded from Thailand (Weerasooriya and Saunders 2010), viz. *M.alba* Ridl., *M.keithii* Ridl., *M.sirikitiae* Weeras., Chalermglin & R.M.K.Saunders, *M.teysmannii* Scheff., *M.tomentosa* Hook.f. & Thomson, *M.vulpina* C.E.C.Fisch., *M.wangii* Hu and *M.winitii* Craib. Here we report a new species, *M.monocarpa* R.M.K.Saunders & Chalermglin, collected from Surat Thani Province in Peninsular Thailand. Of the eight previously described Thai species, only four (*M.alba*, *M.keithii*, *M.teysmannii* and *M.vulpina*) grow in Peninsular Thailand, although another species, *M.macclurei* Weeras. & R.M.K.Saunders, occurs in Kedah, Peninsular Malaysia (Weerasooriya and Saunders 2005, 2010).

## New species description

### Mitrephora monocarpa

Taxon classificationPlantaeMagnolialesAnnonaceae

R.M.K.Saunders & Chalermglin sp. nov.

urn:lsid:ipni.org:names:60478755-2

http://species-id.net/wiki/Mitrephora_monocarpa

Figs 1 , 2 , 3

#### Diagnosis.

A new species similar to *M.alba* and *M.keithii*, but distinguished by its solitary flowers that are not borne in an inflorescence, its single carpel per flower and single sessile monocarp per fruit.

#### Type.

**Thailand**: In Para Rubber plantation (*Heveabrasiliensis* (Kunth.) Mull. Arg.), Khlong Sok Village, Phanom District, Surat Thani Province, 15 December 2015, *P. Chalermglin 581215* (holotype: BKF [dry and spirit collection]; isotypes: BK, QBG, PSU, K, SING [dry]).

#### Description.

Treelets or small trees, to ca. 5 m, main trunk slender. Young branches glabrous to sparsely pubescent, with short, appressed golden-brown hairs. Leaf laminas 9–13 cm long, 3.5–5 cm wide, length:width ratio 2.5–3.3, ovate to elliptic, papyraceous, glabrous and ± matt adaxially, glabrous abaxially; base obtuse to slightly cuneate; apex acute to slightly acuminate; primary vein glabrous ad- and abaxially; secondary veins 6–8 pairs per leaf, ± inconspicuous, glabrous ad- and abaxially; domatia absent; petioles 3.5–5 mm long, 1.1–1.4 mm in diameter, glabrous. Inflorescence reduced to a single flower. Flower pedicel ca. 4.5 mm long, ca. 1.5 mm in diameter, pubescent. Sepals ca. 2.5 mm long, ca. 2 mm wide, broadly ovate, pubescent abaxially. Outer petals 11.5–12.5 mm long, 7–8 mm wide, cream-coloured (turning yellowish in late-stage flowers), ovate, apex acute, margin not undulating with age, pubescent abaxially, sparsely pubescent adaxially. Inner petals ca. 9.5 mm long, ca. 3 mm wide apically, ca. 2 mm wide at claw, pale pink with whitish margins and near point of apical connivance (turning yellowish in late-stage flowers), clawed-triangular, hairs non-glandular. Stamens 0.9–1 mm long, 0.7–0.8 mm wide. Carpels solitary per flower, ca. 1.4 mm long; ovary ca. 0.9 mm long, ca. 0.5 mm wide, densely pubescent; stigma ca. 0.5 mm long, ca. 0.9 mm wide; ovules ca. 9 per carpel, in two columns. Monocarp solitary per fruit, ca. 52 mm long, ca. 32 mm in diameter, ellipsoid, smooth, without longitudinal ridge; stipe absent. Fruit pedicel ca. 6 mm long, ca. 4 mm in diameter. Seeds ca. 9 per monocarp, size unknown (fruiting specimen not preserved).

**Figure 1.**
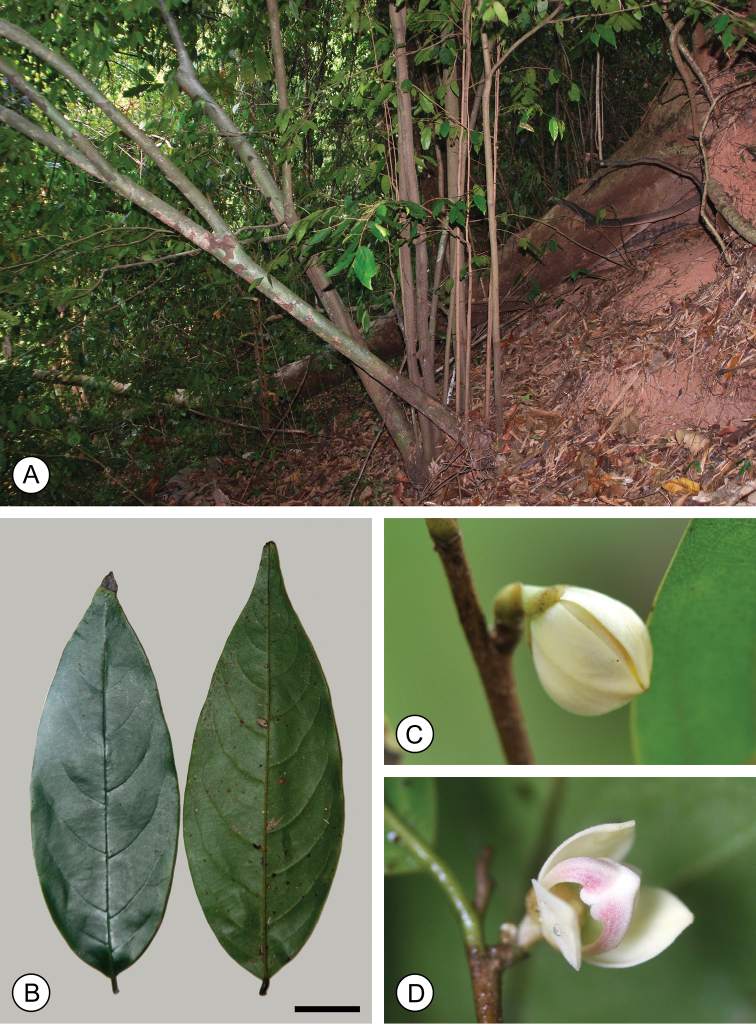
*Mitrephoramonocarpa* sp. nov. (*P. Chalermglin 581215*). **A** Habit **B** leaves (left: adaxial; right: abaxial) **C** flower bud **D** young flower, shortly after separation of outer petals. Scale bar: 2 cm. Photos: P. Chalermglin.

#### Phenology.

**Figure 2.**
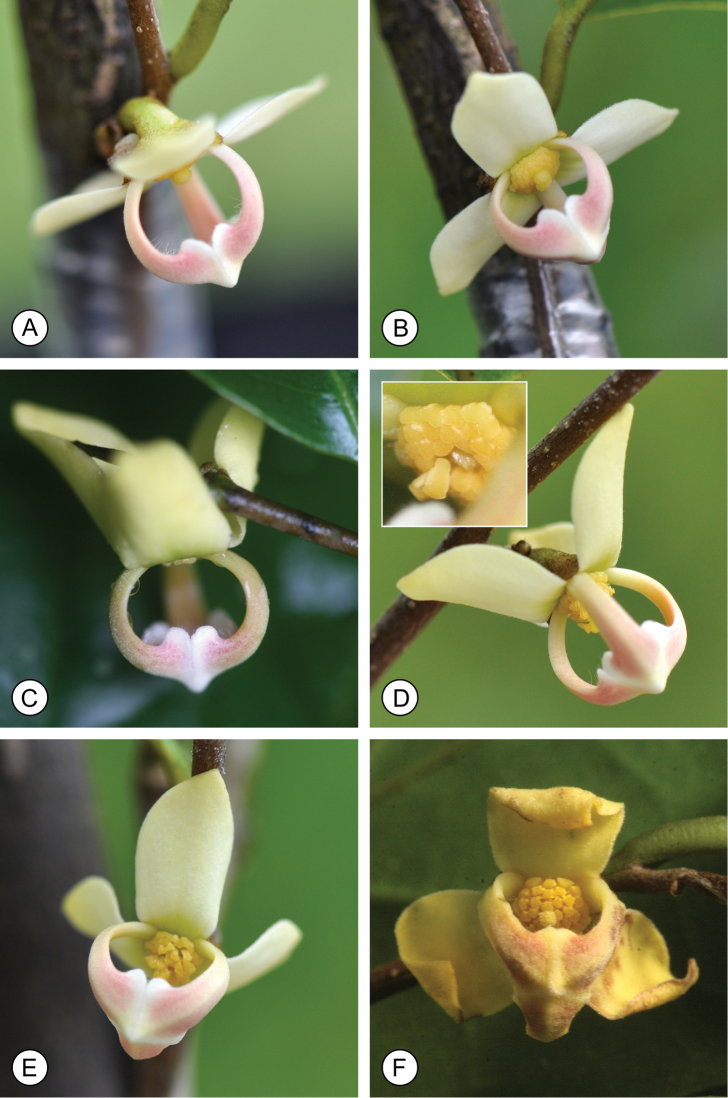
*Mitrephoramonocarpa* sp. nov. (*P. Chalermglin 581215*). **A, B** Pistillate-phase flowers **C–E** staminate-phase flowers (insert in **D** shows abscised stamens suspended by tracheary elements in the xylem) **F** late-stage flower, with petals turning yellow. Photos: P. Chalermglin.

Flowering was observed in June, July and December. As with all hermaphroditic-flowered Annonaceae species (Pang and Saunders 2014), *M.monocarpa* is protogynous. The flowers undergo visible change associated with the switch between the pistillate and staminate function: the outer petals are held at right-angles to the floral axis in pistillate-phase flowers (Fig. 2A, B), but become reflexed during the staminate phase (Fig. 2C–E). The stamens partially abscise as the thecae dehisce and ultimately remain suspended from the floral torus by their tracheary elements (inset in Fig. 2D; cf. Endress 1985). As the flower ages, the petals of both whorls begin to turn yellow and wilt (Fig. 2F) before abscising. Fruiting was observed in December.

#### Distribution and habitat.

*Mitrephoramonocarpa* is only known from the type collection cited above, from a tropical rain forest over limestone, ca. 250 m elevation.

#### Etymology.

The specific epithet reflects the fact that the flower has only a single carpel and, hence, the fruit consists of a solitary monocarp.

**Local name**: Phrom Phanom.

#### Discussion.

The phylogenetic affinities of *M.monocarpa* are currently unknown, although morphologically it resembles two species, *M.alba* and *M.keithii*, with which it is broadly sympatric in Peninsular Thailand: all three species have a sparsely hairy indument on the twigs and leaves and comparatively small flowers. *Mitrephoramonocarpa* is clearly distinguished from these species, however, as the flowers have only one carpel: *M.alba*, in contrast, has ca. 16 carpels per flower and *M.keithii* has 12–14 (Weerasooriya and Saunders 2010). Carpel number is variable in other species in the genus, although the smallest number previously recorded was four (in the Indochinese species *M.calcarea* Diels ex Weeras. & R.M.K.Saunders, the Bornean species *M.kostermansii* Weeras. & R.M.K.Saunders, the Philippine species *M.lanotan* (Blanco) Merr. and the Sumatran species *M.rufescens* Ridl.; Weerasooriya and Saunders 2010).

**Figure 3.**
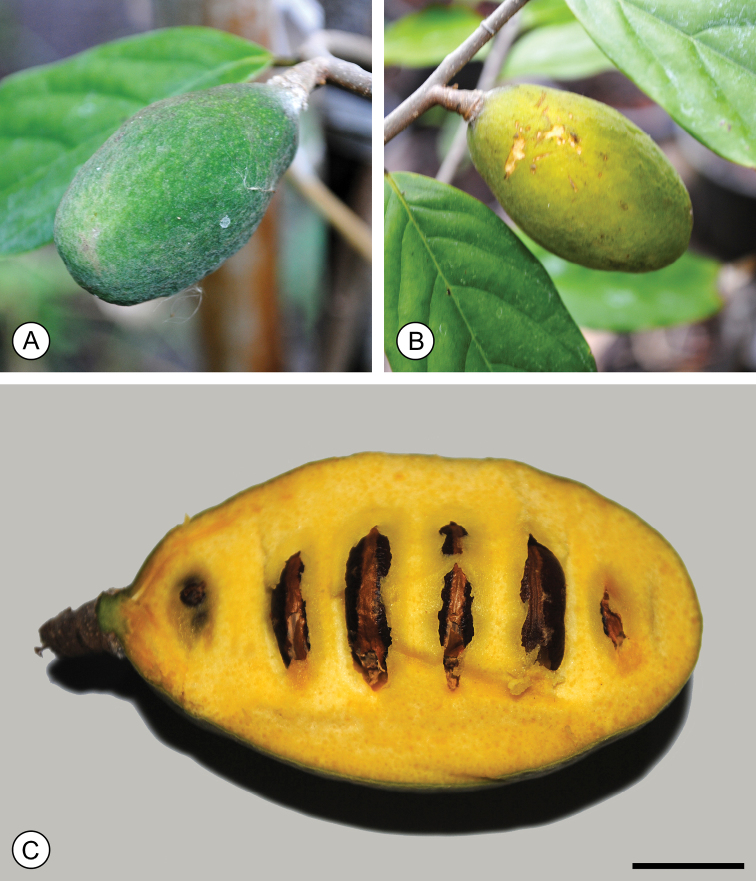
*Mitrephoramonocarpa* sp. nov. (*P. Chalermglin 581215*). **A** Immature fruit, composed of a single monocarp **B** partially mature fruit, with the exocarp turning yellow **C** dissected fruit, showing multiple seeds. Scale bar: 1 cm. Photos: P. Chalermglin.

The fruits of *M.monocarpa* are easily distinguished from those of *M.alba* and *M.keithii* as they consist of only one monocarp (Fig. 3A, B); this monocarp is furthermore sessile, whereas those in *M.alba* and *M.keithii* are stipitate, with stipes that are 6–15 mm and 3–3.5 mm long, respectively.

*Mitrephoramonocarpa* also resembles the Bornean species *M.uniflora* Weeras. & R.M.K.Saunders in possessing solitary flowers (Weerasooriya and Saunders 2010), presumably as a result of the evolutionary reduction of the thyrsoid inflorescence, evident in other *Mitrephora* species.

##### Key to *Mitrephora* species in Thailand

**Table:** 

1a	Outer petals 37.5–53.5 × 22–53 mm; inner petals 28–43 × 22–41 mm; monocarps with 13–21 seeds.	**2**
2a	Leaf laminas glossy adaxially, with 8–11 pairs of secondary veins; flower pedicels 18–27 mm long; sepals 13.5–15.5 × 14–19.5 mm; outer petals 44–53.5 × 41–53 mm; inner petals 37–43 × 36.5–41 mm	***Mitrephorasirikitiae* Weeras., Chalermglin & R.M.K.Saunders**
2b	Leaf laminas matt adaxially, with 11–13 pairs of secondary veins; flower pedicels 10–15.5 mm long; sepals 7.5–10.5 × 7.5–11 mm; outer petals 37.5–40 × 22–29.5 mm; inner petals 28–32 × 22–24.5 mm	***Mitrephorawinitii* Craib**
1b	Outer petals 9.5–34.5 × 6.5–19 mm; inner petals 6–19 × 3–12.5 mm; monocarps with 4–10 seeds	**3**
3a	Young branches sparsely pubescent	**4**
4a	Flowers and fruits with a solitary carpel; monocarp sessile	***Mitrephoramonocarpa* R.M.K.Saunders & Chalermglin, sp. nov.**
4b	Flowers and fruits with 12–16 carpels; monocarps stipitate	**5**
5a	Flower pedicels 10–16 mm long; sepals 1.5–2.5 mm long; outer petals white, 10.5–15.5 mm wide; inner petals white with pink/purple margins, 9–14.5 × 5.5–11 mm; monocarps warty, with longitudinal ridge; stipes 6–15 mm long	***Mitrephoraalba* Ridl.**
5b	Flower pedicels 4.5–9 mm long; sepals 3–4 mm long; outer petals yellow, 6.5–9.5 mm wide; inner petals yellow with pink margins, 7.5–9.5 × 4.5–6 mm; monocarps smooth, without longitudinal ridge; stipes 3–3.5 mm long	***Mitrephorakeithii* Ridl.**
3b	Young branches densely pubescent	**6**
6a	Inflorescence rachides with internodes that elongate, bearing > 3 flowers; 36–40 carpels per flower; monocarps not glaucous	***Mitrephoravulpina* C.E.C.Fisch.**
6b	Inflorescence rachides with internodes that do not elongate, bearing < 3 flowers; 8–17 carpels per flower; monocarps glaucous	**7**
7a	Leaf laminas densely pubescent abaxially; sepals 5–9 × 5–9 mm; fruit pedicels densely pubescent; monocarps globose.	***Mitrephoratomentosa* Hook.f. & Thomson**
7b	Leaf laminas subglabrous to sparsely pubescent abaxially; sepals 1.5–4 × 2–5.5 mm; fruit pedicels sparsely pubescent; monocarps obovoid or oblong	**8**
8a	Leaf laminas matt adaxially, sometimes with domatia at axils of secondary veins abaxially; inner petals cream, 6–12.5 mm long, densely pubescent abaxially; 10–14 carpels per flower; fruit pedicels 15–39 mm long, sparsely pubescent; monocarps without longitudinal ridge, densely pubescent	***Mitrephorateysmannii* Scheff.**
8b	Leaf laminas glossy adaxially, without domatia; inner petals purplish, 11–19 mm long, sparsely pubescent abaxially; 8–10 carpels per flower; fruit pedicels 10–16 mm long, densely pubescent; monocarps with longitudinal ridge, sparsely pubescent	***Mitrephorawangii* Hu**

## Supplementary Material

XML Treatment for Mitrephora monocarpa
